# A Septicemic Child due to a Novel Streptococcal Species, *Streptococcus gwangjuensis*

**DOI:** 10.1155/crdi/7886001

**Published:** 2025-11-07

**Authors:** Benjawan Comhangpol, Sawita Srisawat, Naowarat Wangnadee, Kittiphong Matvises, Phatsaraporn Sirisa, Kotchaphan Sonoutha, Anusak Kerdsin

**Affiliations:** ^1^Roi Et Hospital, Roi Et, Thailand; ^2^Faculty of Public Health, Kasetsart University, Chalermphrakiat Sakon Nakhon Province Campus, Sakon Nakhon, Thailand

**Keywords:** amplicon-based NGS, febrile neutropenia, *gyrB*, septicemia, *sodA*, *Streptococcus gwangjuensis*

## Abstract

We report a case of septicemia due to *Streptococcus gwangjuensis* in an immunocompromised 8-year-old child. The final diagnosis was febrile neutropenia associated with acute myeloid leukemia and septicemia. The patient was successfully treated with chemotherapy, granulocyte colony-stimulating factor, leukodepleted packed red cell transfusions, piperacillin/tazobactam, and meropenem. *S. gwangjuensis* was identified using *sodA* and *gyrB* amplicon-based next-generation sequencing. The isolate was susceptible to all β-lactam antibiotics.

## 1. Introduction

*Streptococcus* is a gram-positive cocci and catalase-negative belonging to the family S*treptococcaceae* in the order *Lactobacillales* [1]. Streptococci can be disease-causing in humans and in many animal species, commensals, and opportunistic [1]. Streptococci could be distinguished as pyogenic streptococci and nonpyogenic streptococci [1]. Nonpyogenic streptococci include mostly alpha-hemolytic as well as beta-hemolytic species. Most of the nonpyogenic streptococci are known as viridans group streptococci (VGS) [1]. VGS composed five species groups designated the *S. mitis* group, the *S. anginosus* group, the *S. salivarius* group, the *S. mutans group*, and the *S. equinus* group [1]. The VGS are commensal inhabitants in the oral, respiratory, and skin in the human host, and also opportunistic pathogens [1].

The *Streptococcus mitis* group (mitis group) is commensal, commonly found in the gastrointestinal and genitourinary tracts, oral mucosa, and on tooth surfaces [1]. These bacteria can cause a variety of infections, including sepsis, meningitis, endocarditis, dental caries, and abscesses [1]. They also play a significant role in exacerbating influenza infections, particularly in immunocompromised individuals [2]. This group includes approximately 20 species with validly published names [3]. Some species in the mitis group, such as *S. pseudopneumoniae*, *S. mitis*, *S. oralis*, and *S. infantis*, are known opportunistic pathogens [1].

*Streptococcus gwangjuensis* is a proposed novel species within the genus *Streptococcus* and is classified under the mitis group of VGS. It was first described in 2019 as *S. gwangjuense* and then corrected as *S. gwangjuensis* in 2004 [4, 5]. It was originally isolated from a human pericoronitis lesion in the Republic of Korea [4]. Here, we describe the first reported case of *S. gwangjuensis* infection in a child in Thailand.

## 2. Case Presentation

An 8-year-old boy was admitted to a hospital in Roi Et Province, Northeastern Thailand, in February 2021, with a fever lasting 10–14 days. He had a medical history of acute myeloid leukemia (AML) and had undergone Phase 1 chemotherapy involving granulocyte colony-stimulating factor (G-CSF) at a tertiary hospital in Khon Kaen Province in November 2020. Upon admission, his physical examination revealed a temperature of 37.9°C, a pulse rate of 120 beats/min, a respiratory rate of 26 breaths/min, and a blood pressure of 100/60 mmHg. The patient was admitted to the pediatric ward, had no indwelling vascular device, and underwent echocardiography. In addition, he had no record of a history of oral cavity maintenance. Laboratory results showed a hemoglobin level of 6.2 g/dL, a hematocrit of 18.8%, and a white blood cell count of 1300 cells/μL. The initial diagnosis was febrile neutropenia.

A bacterial isolate (STC26) was obtained from the patient's blood and identified as the *Streptococcus mitis* group at the hospital laboratory using conventional biochemical tests [1]. The isolate was then sent to the Public Health Microbiology Laboratory, Faculty of Public Health, Kasetsart University, for confirmation. It was identified using *sodA* and *gyrB* amplicon-based next-generation sequencing with the Illumina platform because these two genes are highly accurate to identify species of VGS [6–9]. Sequencing depth of amplicon-based next-generation sequencing is 100X for both *sodA* and *gyrB*. Almost nucleotide identity of the strain STC26 was 98.05% for *sodA* and 99.76% for *gyrB* with *S. gwangjuensis* type strain (ChDC B345^T^), that greater than other species of the mitis group. Therefore, the isolate was identified as *S. gwangjuensis*. Antimicrobial susceptibility was conducted according to the M100 33rd edition of the CLSI guidelines [10]. The isolate STC26 was susceptible to penicillin (MIC ≤ 0.12 mg/mL) and ampicillin (MIC ≤ 0.25 mg/mL) using the Sensititre™ Complete Automated AST System (Thermo Scientific, USA). Disc diffusion tests indicated susceptibility to vancomycin, clindamycin, erythromycin, and ceftriaxone.

The final diagnosis was *S. gwangjuensis* bacteremia and sepsis in the context of AML and resultant neutropenia. The patient was treated with chemotherapy, G-CSF, leukocyte-depleted red cell transfusions, packed red cell transfusions (3 units), piperacillin/tazobactam, and meropenem. He was discharged in February 2021 after 15 days of hospitalization. The hospital followed up with the patient after discharge seven days later, and the patient recovered well.

## 3. Discussion

*S. gwangjuensis* was proposed in 2019 as a novel species of the genus *Streptococcus* based on average nucleotide identity and genome-to-genome distance values for bacterial discrimination [4]. It is closely related to *S. mitis* and *S. pseudopneumoniae*, both members of the mitis group [4]. To date, only one other human case, involving a male with a pericoronitis lesion in the Republic of Korea, has been reported. The isolate from that case is a type strain, ChDCB345^T^ (also known as KCOM1679^T^ or JCM33299^T^) [4, 5].

In this study, we report a case of *S. gwangjuensis* infection in an immunocompromised child. This revealed epidemiological information about *S. gwangjuensis* can cause disease in a child in the current study and an adult in a previous study [4]. Although we could not determine the exact route of infection, it is possible that the bacteria, which commonly inhabit the oral cavity, gastrointestinal, and genitourinary tracts [1], invaded the bloodstream. Further studies on *S. gwangjuensis*, including its ecology, virulence, and pathogenesis, are needed. The isolate in this case was susceptible to β-lactam antibiotics, including meropenem, which was used for treatment. However, enhanced antimicrobial stewardship is necessary to limit unnecessary antibiotic use and preserve empirical treatment options. A study in the United States revealed that cumulative susceptibility rates of VGS from 2010 to 2020 were high for ceftriaxone (96.0% to 100%), clindamycin (81.3% to 84.5%), and vancomycin (99.7% to 100%) [11]. Specifically, *S. mitis*, *S. oralis*, and nonspeciated isolates showed penicillin susceptibility rates of 71.0%, 80.9%, and 86.3%, respectively [11]. In Japan, a reduction in carbapenem susceptibility was observed in *S. mitis/S. oralis* clinical isolates [12].

Accurate identification of VGS, including the mitis group, is crucial for assessing clinical significance, guiding appropriate antimicrobial therapy, and conducting epidemiological surveillance. Traditional biochemical tests and 16S rDNA sequencing are insufficient for discriminating species within the mitis group and VGS [7, 8]. Advanced techniques like matrix-assisted laser desorption ionization-time of flight mass spectrometry (MALDI-TOF MS), multilocus sequence analysis (MLSA), whole-genome sequencing (WGS), and sequencing of genes such as *sodA, rpoB, ddl, recN, gyrB*, and *groESL* have demonstrated high discriminatory power for identifying VGS species, including those in the mitis group [3, 6–9, 13–18]. However, MALDI-TOF MS requires expensive instrumentation and an updated database, while WGS and MLSA are not feasible in routine laboratories due to cost, complexity, and time constraints, as well as the need for bioinformatics expertise.

As a practical alternative, identification based on *sodA* sequencing is recommended [8]. *sodA* sequencing is a reliable and straightforward method for accurate species identification within the mitis group [9]. However, a study by Galloway-Peña et al. (2014) indicated that *sodA* sequencing alone might be insufficient for precisely identifying certain species within the mitis group, including *S. oralis*, *S. mitis*, *S. australis*, and *S. infantis* [7]. In this study, we used both *gyrB* and *sodA* sequencing to accurately identify *S. gwangjuensis*. Amplicon-based next-generation sequencing is a cost-effective alternative to Sanger sequencing, as it allows for the simultaneous sequencing of multiple amplicons. This method has been successfully applied to identify various pathogens described elsewhere [19–21].

In conclusion, we report a case of septicemia due to *S. gwangjuensis* in an immunocompromised child. The isolate was susceptible to all tested antimicrobial agents. The combination of *sodA* and *gyrB* sequencing proved to be a reliable identification method.

## Data Availability

The manuscript includes all data related to this case report. Additional clinical information may be available from the corresponding author, provided it complies with confidentiality regulations and is requested reasonably. The *gyrB* and *sodA* sequences of the *S. gwangjuensis* strain STC26 in the study were deposited in GenBank with accession numbers PV996940 and PV996941, respectively.
